# Editorial: DNA and RNA Metabolism Meet at Chromatin to Control Genome Stability

**DOI:** 10.3389/fgene.2016.00067

**Published:** 2016-04-27

**Authors:** Alessandra Montecucco, Giuseppe Biamonti

**Affiliations:** Consiglio Nazionale delle Ricerche, Istituto di Genetica MolecolarePavia, Italy

**Keywords:** genome stability, DNA Replication, RNA metabolism, DNA damage response, histone modifications

The integrity of the genome is continuously challenged by endogenous and exogenous DNA damaging agents and by lesions arising during DNA replication and transcription. To prevent the severe biological consequences that may arise from DNA injuries, cells have evolved an intricate network of genome surveillance mechanisms, collectively designated as DNA damage response (DDR). In addition to damage-specific repair machineries, perturbations in the structure and continuity of the DNA molecule trigger checkpoint pathways that delay or arrest cell-cycle progression thus providing more time for repair mechanisms. Moreover, checkpoint pathways coordinate DNA repair with DNA replication and transcription. Functional alterations in any of these processes and in their crosstalk may result in uncontrolled cell proliferation or programmed cell death, two opposite events that are harmful both at the cellular and organismal level.

DNA in the eukaryotic genome folds, for most part, into the canonical B-form and is packaged into chromatin that is the substrate of all DNA transactions. Complex DNA secondary structures (G-quadruplexes), hybrid DNA/RNA structures (R-loops), and changes in chromatin architecture induced by reversible histone post-translational modifications, incorporation of histone variants and ATP-dependent chromatin remodeling enzymes, occur in response to external and cellular cues and affect vital processes such as regulation of gene expression, DNA replication, and repair. Histone modifications are best understood for their effects on transcription, but it is becoming increasingly evident that they also function in the DDR, where chromatin reorganization is required to allow access of repair proteins to DNA lesions. However, we are still far from a clear and comprehensive picture of chromatin dynamics in relation to DDR. Finally, recent data implicate splicing factors, small non-coding RNAs, and components of the RNA interference machinery in chromatin organization both in unstressed cells and in response to DNA damage.

The articles in this special issue provide a view of the complex network of interactions between DNA and RNA metabolism that contribute to genome stability in eukaryotic cells.

DNA replication is an extremely risky process. Cea et al. address the problem of complex secondary structures such as G-quadruplexes (G4) that can constitute an obstacle to DNA and RNA metabolism. The resolution and replication of structured DNA is therefore crucial to prevent genetic and epigenetic instability across the genome. The authors focus on how G4 DNA is resolved during replication, and how its successful bypass requires the coordinated activity of single stranded DNA (ssDNA) binding proteins, helicases, and specialized DNA polymerases. G-rich elements with potential to form G-quadruplex structures have been described in most of the replication origins in mouse and human cells (Besnard et al., 2012). In their intriguing perspective Lombraña et al. propose that R-loops form at CpG island promoters during transcription and can contribute to DNA replication origin specification just activating the formation of G4 structures that recruit the Origin Recognition Complex. In this hypothesis R-loop dysregulation at CpG islands associated with promoter-origin regions might contribute to the phenotype of DNA replication abnormalities and to the loss of genome integrity detected in cancer cells. R-loops can also be the pathological outcome of replication and transcription collisions. Brambati et al. review the current knowledge on replication and transcription conflicts in eukaryotes, their consequences on genome instability and the pathways involved in their resolution, whose understanding is relevant to clarify the molecular basis of cancer and neurodegenerative diseases. R-loop structures also form at telomeres in yeast and in mammalian cells where the TERRA (telomeric repeat-containing RNA) transcripts can base pair with their template DNA. Cusanelli and Chartrand discuss the recent developments on TERRA's role in telomere biology and genome integrity, and its implication in cancer.

A growing body of evidence suggests that different types of non-coding RNAs (ncRNAs) can directly affect chromatin conformation, and in this way may impact transcription and splicing, as well as promote the activation of DDR. Recently, it has been suggested that small ncRNAs generated by the RNA interference machinery are involved in DDR signaling and homology-mediated DNA repair. Francia reviews the experimental evidence that ncRNAs act at the genomic loci from which they are transcribed to modulate chromatin organization, DDR and DNA repair.

Mounting evidence supports the idea that RNA-binding proteins involved in different steps of mRNA life can affect genome stability programs (Montecucco and Biamonti). In this issue Naro et al. focus on the non-canonical role of splicing factors and DNA repair proteins as gatekeepers of genome stability. In fact, several splicing factors have been recently shown to play direct function in DNA repair, beyond their expected splicing activity, and at the same time evidence is emerging that DNA repair proteins can act as post-transcriptional regulators of gene expression. This finding suggests the existence of a tight interplay between splicing regulation and DNA safeguard mechanisms ensuring genome stability.

Among reversible protein modifications, ubiquitination is broadly implicated in cellular functions. In particular, signaling processes mediated by ubiquitin are crucial for the cellular response to DNA double-strand breaks (DSBs). Citterio discusses the current knowledge of how ubiquitin-mediated signaling at DSBs is controlled by deubiquitinating enzymes (DUBs) with particular emphasis on histone DUBs and their recent involvement in stem cell biology and cancer.

## Author contributions

All authors listed, have made substantial, direct, and intellectual contribution to the work, and approved it for publication.

### Conflict of interest statement

The authors declare that the research was conducted in the absence of any commercial or financial relationships that could be construed as a potential conflict of interest.
